# Electrostatically Biased Binding of Kinesin to Microtubules

**DOI:** 10.1371/journal.pbio.1001207

**Published:** 2011-11-29

**Authors:** Barry J. Grant, Dana M. Gheorghe, Wenjun Zheng, Maria Alonso, Gary Huber, Maciej Dlugosz, J. Andrew McCammon, Robert A. Cross

**Affiliations:** 1 Department of Chemistry and Biochemistry, Center for Theoretical Biological Physics and Howard Hughes Medical Institute, University of California–San Diego, La Jolla, California, United States of America; 2 Centre for Mechanochemical Cell Biology, Warwick Medical School, University of Warwick, Coventry, United Kingdom; 3 Department of Physics, University at Buffalo, Buffalo, New York, United States of America; 4 Interdisciplinary Centre for Mathematical and Computational Modelling, University of Warsaw, Warsaw, Poland; 5 Department of Pharmacology, University of California–San Diego, La Jolla, California, United States of America; UC Davis, United States of America

## Abstract

The minimum motor domain of kinesin-1 is a single head. Recent evidence suggests that such minimal motor domains generate force by a biased binding mechanism, in which they preferentially select binding sites on the microtubule that lie ahead in the progress direction of the motor. A specific molecular mechanism for biased binding has, however, so far been lacking. Here we use atomistic Brownian dynamics simulations combined with experimental mutagenesis to show that incoming kinesin heads undergo electrostatically guided diffusion-to-capture by microtubules, and that this produces directionally biased binding. Kinesin-1 heads are initially rotated by the electrostatic field so that their tubulin-binding sites face inwards, and then steered towards a plus-endwards binding site. In tethered kinesin dimers, this bias is amplified. A 3-residue sequence (RAK) in kinesin helix alpha-6 is predicted to be important for electrostatic guidance. Real-world mutagenesis of this sequence powerfully influences kinesin-driven microtubule sliding, with one mutant producing a 5-fold acceleration over wild type. We conclude that electrostatic interactions play an important role in the kinesin stepping mechanism, by biasing the diffusional association of kinesin with microtubules.

## Abstract 2

An electrostatic field rotates, slides, and guides the kinesin head to bind the microtubule at a site a short distance ahead, thus determining the direction of movement of the motor.

## Author Summary

Animal and plant cells contain a molecular-scale “railway” network, in which the tracks, called microtubules, radiate out from the cell centre and locomotive proteins, called kinesins, haul their molecular cargoes along the microtubule tracks. This railway system transports many different cargoes to where they are needed, so it is crucial for the cell's organization and function. Breakdowns in this transport system can cause diseases like Alzheimer's, and drugs that temporarily halt transport make powerful anti-cancer agents. Precisely how kinesin motor proteins move along their microtubule tracks is an important question in biology. We know that some kinesins have twin “heads” that alternately bind to and step along microtubules in a coordinated walking action. But more usually, kinesins have only one head. How single-headed kinesins produce force and movement is poorly understood. In this study, we address this question and show that electrical attraction between single kinesin heads and microtubules is a critical factor deciding the direction of movement: each time the head approaches a microtubule, it slides forwards by the electrical attraction between the engine and the track.

## Introduction

Kinesins form a large family of ATP dependent microtubule-based motor proteins. At least 14 sub-families have been identified [1]–[3], the members of which play a wide variety of roles in intracellular transport, including vesicle and organelle transport, cytoskeletal reorganization, and chromosome segregation [4]. Underpinning these diverse activities is a coupling of ATP turnover, microtubule bind-release cycles, and unidirectional mechanical motion. Several features of the mechanisms by which kinesins generate force and movement are known, but many uncertainties remain. Kinesin-1, the best studied kinesin, has twin heads and moves towards microtubule plus ends using a head-over-head walking action that can do work against loads of up to ∼7 pN [5],[6]. Importantly however, the minimal motor domain of kinesin-1 is a single head [7]. Teams of single kinesin-1 heads can drive directional microtubule sliding, with each head in the team contributing intermittent impulses of force and motion. Less is known about this mechanism, by which individual kinesin heads generate directional force.

**Figure 1:**
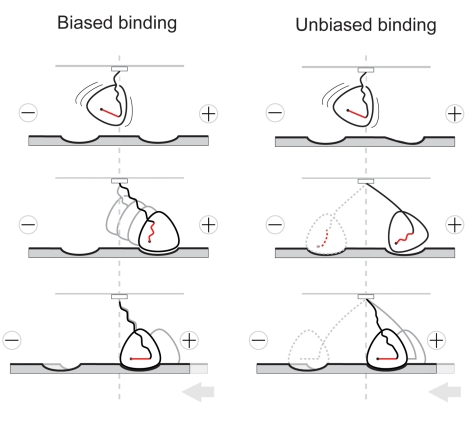
Biased binding and unbiased binding frameworks for the kinesin minimal motor domain mechanism. (Left) In biased binding models, the motor domain diffuses on a tether and diffusion-to-capture is directionally biased. (Right) In models with unbiased binding, diffusion-to-capture occurs with equal probability in both directions and progress is due to a subsequent conformational change. Conformational changes that follow binding in the progress direction contribute useful force, conformational changes that follow binding in the antiprogress direction do not.

Broadly, two different classes of model have been proposed for the mechanical cycle by which kinesin heads generate force and movement—biased binding models and unbiased binding models. In biased binding models, the motor domain diffuses back and forth on a spring-like tether, using thermal energy from the bath to stretch out the tether, locking on to the track at a moment when the spring is stretched out in the progress direction, and then maintaining its grip on the track whilst the spring relaxes. Biased binding models like this (Figure 1, left) are sometimes referred to as thermal ratchets [8]. The classic example of this type of model is the Huxley 1957 [9] model for the myosin crossbridge. In biased binding models, most of the ground gained is due to directionally biased diffusion-to-capture. The directionally biased capture event is envisaged to involve or trigger a directional conformational change and one or more coupled chemical steps, but the conformational change is negligibly small compared to the stepping distance. By contrast, models with unbiased binding (Figure 1, right) envisage that the probability of binding of kinesin heads to microtubules is the same in both directions and that directional stepping is entirely due to one or more directional conformational changes that occur after the motor has engaged with its binding site. Current controversies over the role of neck linker docking in the kinesin cycle relate to this same dichotomy. Neck linker docking is a conformational change that is clearly important in the kinesin mechanism [10], but whether neck linker docking can do appreciable work remains uncertain. The results of molecular dynamics simulations argue that substantial work could be done [11]. On the other hand, measurements of the free energy difference between the docked and undocked neck linker indicate ∼5 pN nm [12], suggesting that neck linker docking could not do the work necessary to account for kinesin's ability to step ∼8 nm against a ∼7 pN load.

Since conformational changes, including neck linker docking, undoubtedly do occur once the kinesin head is attached to the microtubule [13], the key problem is to find out whether a biased binding mechanism contributes appreciably to the kinesin mechanical cycle or whether instead binding is unbiased and the generation of directional force is entirely due to one or more conformational changes that follow microtubule binding.

There is clear evidence that tethered single kinesin heads can develop impulses of directional force and displacement. These step-displacements have been estimated using single molecule optical trapping to be 3–4 nm, and attributed to biased binding [14],[15]. Many theoretical models [16],[17] posit that biased binding occurs and that it is driven by one or more directional sawtooth binding potentials. As yet, however, a specific molecular mechanism is lacking. This is the problem we address in the current work.

It is known for a number of non-motor systems that electrostatic interactions can effectively maneuver associating proteins into a suitable binding configuration, a phenomenon known as electrostatic steering [18],[19]. Formation of the final tightly bound complex from the encounter complex may require internal structural rearrangements as well as more local effects, including dehydration of the binding interface. Electrostatics is known to play a role in the binding of kinesin to microtubules, with roles established for the negatively charged E-hook of tubulin, and for the positively charged K-loop of kinesin, in both the Kif1a (kinesin-3) [20] and kinesin-1 [21] mechanisms, and for charged residues and ionic strength in general [22]. In the present work we have sought to test whether long-range electrostatic guidance might govern not only the rate, but also the approach trajectory, of kinesin-microtubule encounters.

To approach this question, we performed electrostatic calculations and atomistic Brownian dynamics simulations in parallel with in vitro motility assays of electrostatically engineered mutant kinesin motors. Our results demonstrate a strong tendency for long-range electrostatic guidance to enhance kinesin-tubulin association and encounter complex formation. Expanded simulations of kinesin dimers on short sections of microtubule indicate that conserved electrostatic interactions not only enhance association but also enable kinesin heads to bind preferentially to sites lying ahead in the progress direction. We further find that the tethering of two heads in a dimer reduces the search space for binding sites on the microtubule lattice, effectively enhancing directional bias and providing a mechanism to track single microtubule protofilaments. Simulations with a range of subfamily representatives and selected charge neutralizing mutations suggest that different kinesin subfamilies have tailored their electrostatic properties to modulate association rates and the directional bias of the association reaction along the microtubule. We conclude that electrostatic interactions play an important role in kinesin stepping by guiding the biased diffusional association of kinesin with microtubules.

## Results and Discussion

### Comparative Electrostatic Analysis Highlights the Tubulin Binding Site on Kinesin

**Figure 2:**
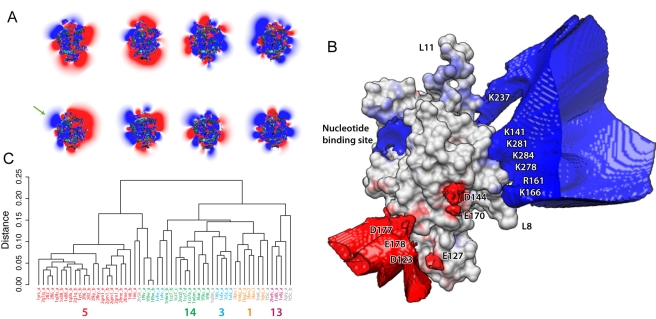
Electrostatic analysis. (A) Surface mapped electrostatic potentials for kinesin family representatives (see Movie S1 for additional mappings). Values are expressed as a color spectrum ranging from +5 kT/e (blue) to −5 kT/e (red). Note, despite the overall diversity in charge distribution, the consistent positive patch (blue) on the rear face of the motor domain (see also Movie S1). (B) Consensus electrostatic potential map of the kinesin family illustrating regions where 80% of structures have a potential of the same sign (see Movie S2 for additional consensus levels). (C) Electrostatic clustering of available kinesin structures. Structures are labeled with their PDB code and colored by sub-family.

Electrostatic calculations of available motor domain crystal structures spanning 11 kinesin sub-families reveal considerable diversity in patterns of surface charge distribution (Figure 2A and Movie S1). Nevertheless, all structures analyzed possess an invariant region of positive potential (blue) in the nucleotide-binding site and over the back face, particularly loop8, loop7, loop12, and alpha5 (including residues R284, K281, R278, K141, K237, R161, and K166). Also apparent are regions of consistent negative potential (red) located near the loop preceding α3 (residues D144 and E170), giving rise to a common underlying asymmetric charge distribution in the kinesin family (Figure 2B and Movie S2).

The conserved positive potential at the nucleotide-binding site reflects the role of this region in coordinating the negatively charged phosphates of ATP. The other conserved region of positive potential spreads across a considerable part of the microtubule-binding surface of the head (Figure 2B), reflecting the established role for this surface in binding to the negatively charged surface of the microtubule. Alanine scanning mutagenesis [22] and limited proteolysis [23] support this view and more recent high-resolution cryoelectron microscopy studies [24],[25] confirm that following microtubule binding this region becomes buried in the microtubule-kinesin interface. Our analysis identifies several further regions of more subtle conservation of positive charge, such as those in the neighborhood of α3 and α6 (including residues R326, K328, D177, E178, and D123). Such regions are not identified with conventional sequence analysis methods [26].

### Electrostatic Interactions Pre-Orient and Accelerate Kinesin-Tubulin Association

**Figure 3:**
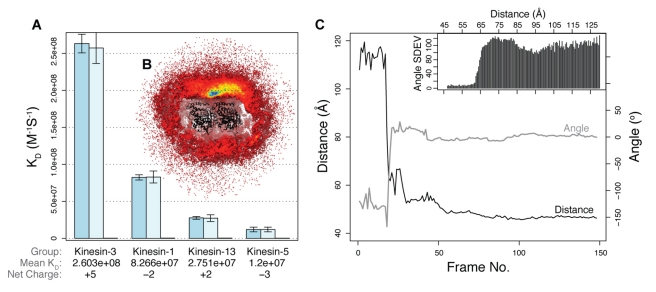
Kinesin-tubulin BD simulations. (A) Subfamily association rates from BD simulations. Two structures from each sub-family were simulated (PDB codes: 1bg2, 1goj, 1i6i, 1vfz, 1ii6, 2gm1, 1v8j, and 1v8k). Error bars represent 95% confidence intervals for the rate determination calculation. Note basal rates (dark bar) were determined in the absence of electrostatic forces for one subfamily representative only. (B) Occupancy maps highlight preferred association sites during BD simulations. Color coded sampling density (occupancy maps) of kinesin-3 about a tubulin heterodimer. Note the single preferred binding site and an apparent preferred path of approach to the bound configuration. (C) Kinesin-tubulin association center-of-mass distance versus relative torsion angle between kinesin and tubulin during successful approach trajectories. The insert plots the standard deviation of the relative torsion angle between kinesin and tubulin at a given separation distance during 200,000 trajectories.

Further comparison and clustering of the calculated electrostatic potentials identified groupings with similar charge distributions (Figure 2C). These results indicated that electrostatic properties are more similar within known sub-families than between sub-families. We selected two representative motor domain structures from four of the largest clusters (representing kinesin-1, 3, 5, and 13 sub-families) as the inputs for our Brownian dynamics simulations. Brownian dynamics simulations were employed to characterize the association process, determine association rates, and investigate the role of long-range electrostatic forces in the association mechanism. Comparison of simulations with and without charges on the motor-domain shows that electrostatic interactions enhance the association rates for all sub-families studied (Figure 3 and Movie S3). As the different motor domains have a range of net charges (+5 to −3), it is unlikely that rate-enhancement arises from nonspecific attraction due to monopole interactions; rather, enhancement of association rates is directly related to the non-uniform charge distribution on kinesin and tubulin. Inspection of BD trajectories clearly shows the steering of the conserved motor domain's positive surface patch toward the negatively charged surface of tubulin (Figure 3B,C), leading to a preferred binding site between tubulin subunits.

Examining successfully associated trajectories indicates that the preferred motor domain approach path lies along a directional trajectory leading from the inter-subunit interface (the alpha-beta junction) toward a single preferred association site located at the beta-alpha intra-heterodimer interface (Figure 3B). The Brownian motion during the approach to binding becomes biased, generating a plus end-directed shearing movement during diffusion-to-capture. Along the preferred approach path, the motor domain's positive patch is predominantly oriented toward the tubulin surface (Figure 2C). This indicates that the motor domain rotates into a specific orientation at an early stage (at a center-to-center distance of ∼60 Å, corresponding to a maximal surface-to-surface separation of ∼15 Å), so that during approach, rotation is constrained such that subsequent motion consists largely of steered translations along the approach trajectory (see also Movie S3). Studies by others on the barnase-barstar system have also characterized significant electrostatic interactions at similar surface-to-surface separation distances [27],[28]. Even at two Debye lengths (∼15 Å at 150 mM ionic strength), interactions will be reduced by about 1/7 compared to contact, which can still yield significant steering effects for highly charged proteins [28]. Simulations with kinesin and tubulin show that at higher ionic strength, electrostatic steering is partially quenched (Figure S4).

### Kinesin Sub-Families Have Distinct Ionic Strength Dependent Association Rates

BD mimics the physical process of diffusional association under the influence of electrostatic interactions. Our simulations indicate that the distinct charge distributions of different kinesin sub-families lead to a range of sub-family-specific association rates (Figure 3A). Kinesin-3 is predicted to have the highest relative association rate (2.6×10^8^ M^−1^ s^−1^) followed by kinesin-1 (8.27×10^7^ M^−1^ s^−1^), kinesin-13 (2.75×10^7^ M^−1^ s^−1^), and kinesin-5 (1.2×10^7^ M^−1^ s^−1^).

Different structures from the same subfamily were found to have very similar association rates reflecting their common charge distributions. Simulations performed under varying salt concentrations showed a similar sub-family trend resulting in decreased association rates at higher ionic strength for all sub-families (see Figure S1).

### Monomeric Motors Have a Preference for Binding the Plus-End of Microtubules

**Figure 4:**
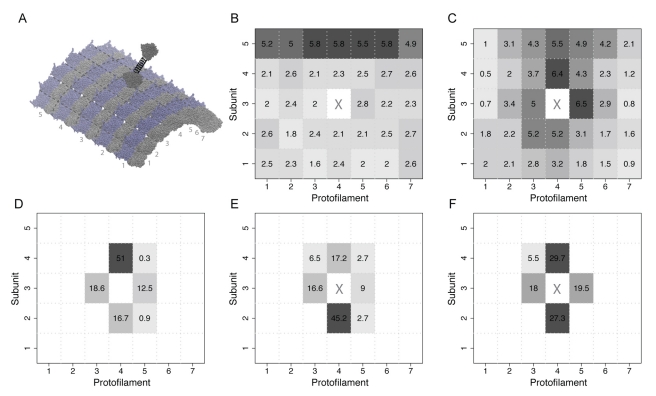
Kinesin-microtubule BD simulations. (A) Simulations utilized a microtubule model consisting of 7 protofilaments each with 5 tubulin heterodimer subunits. For kinesin dimer simulations, a flexible tether was placed between a freely diffusing head and a second immobile microtubule bound head (see methods). (B) Kinesin-1 monomer binding events. Each of the 35 potential binding sites is labeled and colored by the proportion of binding events at a given site. (C) Kinesin-1 un-tethered dimer binding events. Each simulation is commenced with the freely diffusing kinesin head within the tether distance of its immobile partner head. However, no spring constraint is applied. (D) Binding events for tethered kinesin-1 dimers. (E) Binding events for tethered kinesin-14 dimers and (F) uncharged kinesin-14 dimers.

Simulations of monomeric kinesin-1 motor domains interacting with a microtubule fragment consisting of 7 protofilaments, each with 5 tubulin heterodimer subunits (see Figure 4A), indicated that freely diffusing kinesin-1 motor domains have an intrinsic preference for sites at the plus-end of microtubules (Figure 4B). A similar trend was found for other subfamily members, including minus-end directed kinesin-14 (see Figure S2). These simulations indicate that single motor domains have an equal propensity for each tubulin dimer internal to the microtubule lattice. Intriguingly, simulations performed on charge neutralized microtubule lattices have an overall reduced association rate to all sites and do not display a noticeable plus-end preference (see Figure S2). Together these results indicate that electrostatic features present at the plus-end tip of microtubules favor kinesin association. Minoura and colleagues [29] recently showed that charged nanoparticles diffuse one-dimensionally on microtubules and that the amplitude of the diffusional excursions reduces exponentially as the charge increases. It is possible that the provision of extra charge density at microtubule ends represents a general mechanism for targeting the plus-ends of microtubules.

### Kinesin Dimers Show Enhanced Electrostatically Biased Diffusion-to-Capture

Additional simulations were performed on kinesin-1 and kinesin-14 (Ncd) dimers with one freely diffusing head tethered by a spring to a microtubule-bound partner head. Results from these simulations indicate dramatically different binding preferences (Figure 4D–F). Kinesin-1 tethered heads clearly favor the forward plus-end binding site, whilst Ncd tethered heads favor the rearward minus-site. This result indicates an intrinsic or underlying dimer-enhanced directional bias that exists independent of neck-linker [30] or stalk [31],[32] docking and undocking. The majority of binding events occur on the protofilament to which the partner head is attached. Tethering appears to enhance biased binding by reducing the search space for binding sites (Figure 4C–E). Note that surprisingly the same electrostatic interactions and tether geometry that favor the plus-end-biased binding of dimeric kinesin-1 favor the minus-end-biased binding of dimeric kinesin-14. Control simulations without charges returned no apparent directional preference (Figure 4F). Hence, different kinesin subfamilies appear to have tailored their electrostatic properties to not only enhance and modulate association rates but also to influence directionality.

### Simulations Identify Residues That Are Important for Accelerated Association

The core result from our simulation is that conserved electrostatic features on the kinesin head facilitate its electrostatic guidance during the diffusional approach to microtubule binding, leading to a consistent plus-end-directed diffusional motion of the kinesin head in the moments before binding.

**Figure 5:**
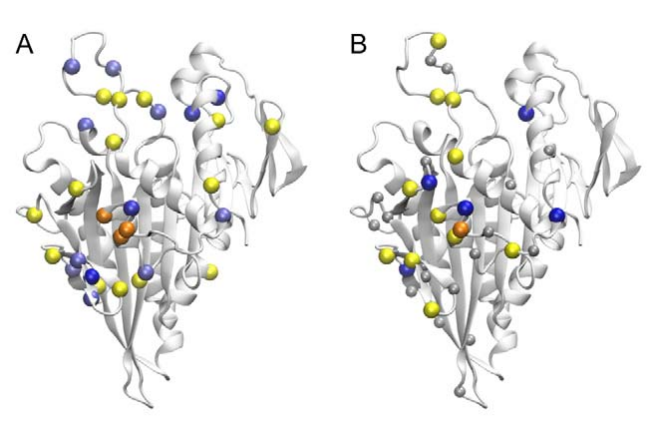
Effects of charge neutralizing alanine mutations mapped to the kinesin-1 structure. (A) Positions whose mutation to alanine decrease (negative: yellow and orange) and increase (positive: light blue and dark blue) calculated ΔΔG_elec_ values. (B) The results of experimental mutagenesis on K_m_MT for microtubule-activated ATPase activity (sites in yellow increase, whilst those in blue decrease K_m_MT); see Woehlke et al.[22] for details.

The simulations allow us to examine the roles of particular residues (on both tubulin and kinesin) in forming the field responsible for this directionally biased diffusion-to-capture. We analyzed the effects of charge-neutralizing mutations on the rate constants of association using BD simulations and the recently developed transient complex approach (see Materials and Methods). By definition the transient complex includes the final bound conformations from successful BD trajectories. We use the ensemble of transient complex configurations to calculate the average electrostatic interaction energy (ΔG_elec_) and the electrostatic interaction energy compared to wild-type (ΔΔG_elec_) (Table 1 and Figure 5).

**Table 1:** The effect of charge neutralizing kinesin mutations on ΔG_elec_ and ΔΔG_elec_ highlight sites important for kinesin-tubulin association.

Mutation	ΔG_elec_* (kJ/mol)	ΔΔG_elec_ (kJ/mol)
R284A	4.915	12.528
K281A	2.872	10.485
N263R	2.585	10.198
R278A	1.72	9.333
K313A	1.081	8.694
K141A	0.663	8.276
K237A	0.269	7.882
R161A	−1.248	6.365
K166A	−2.281	5.332
R321A*	−3.677	3.936
K68A	−3.802	3.811
R203A	−3.897	3.716
K240A	−4.144	3.469
K44A	−4.216	3.397
K252A	−4.287	3.326
K226A	−5.625	1.988
K150A	−5.772	1.841
K131A	−6.329	1.284
K213A	−6.404	1.209
K323A*	−6.506	1.107
K32A	−6.615	0.998
R25A	−6.683	0.93
K159A	−6.833	0.78
K28A	−6.883	0.73
D147A	−8.367	−0.754
D27A	−8.668	−1.055
D249A	−8.741	−1.128
E250A	−8.923	−1.31
E270A	−8.979	−1.366
E170A	−9.123	−1.51
D288A	−9.18	−1.567
E236A	−9.255	−1.642
H156A	−9.27	−1.657
E244A	−9.547	−1.934
L317R	−9.622	−2.009
D158A	−9.805	−2.192
E199A	−9.886	−2.273
E170K	−10.907	−3.294
D279A	−11.003	−3.39
E311A	−11.182	−3.569
D144K	−11.389	−3.776
E309A	−12.094	−4.481
E170A/D144A	−12.651	−5.038
E157A	−13.974	−6.361

The specific predictions made by our simulations about the effects of mutations allow us to test the reliability of our simulations by mutating these residues in the real-world proteins. Computationally, each surface exposed charged residue on kinesin-1 was mutated to alanine and the effect on predicted relative association rates monitored (Table 1). Figure 5A displays these results in relation to the crystallographic structure of kinesin-1 (PDB code: 1bg2). Note the prominent effect of mutations on the rear face of the motor domain. In contrast, mutation of residues on the front face of the motor domain was found to have little impact. Rear positions with a significant influence include those residues contributing to the conserved positive potential patch (i.e., residues R284, K281, R278, K141, K237, R161, and K166, all of which are ranked highly in Table 1). Additional positions in α6 (such as K313, R421, E309, and E311) and β1c (K44) along with the loop before α3 (D144 and E170) were also found to have a significant influence. Also shown in Figure 5 are the published results of experimental alanine scanning mutagenesis by Woehlke and colleagues [22]. Note the excellent correspondence to the results of the Woehlke study, which measured the effects of alanine substitutions on the ATPase and motor activity of kinesin, with the sites highlighted in the current study as influencing association rates and electrostatically guided diffusion-to-capture. Both our calculations and these earlier experiments indicate that substitution of positive residues on the microtubule binding face of kinesin decreases, whilst substitution of negative residues increases association rates.

Mutations that decrease the association rate do so by neutralizing the conserved electrostatic features essential for electrostatic steering. We obtained the largest decreases in the binding rate (of ∼2.3×10^7^ M^−1^ s^−1^) for sites including R284A, K281A, and other contributors to the invariant rear positive potential patch. Association rates could be enhanced (up to a value 7.15×10^7^ M^−1^ s^−1^ for N263R and E170A/D144A) by substituting residues from subfamilies that have an enhanced association rate. A number of control mutants (including D177A/E178A) were also examined and found to yield similar rates to the wild-type complex (8.19×10^7^ M^−1^ s^−1^). Note that D177A and E178A were selected as controls as these residues have a similar proximity to the putative tubulin-binding site as E170A and D144A but were not highlighted by electrostatic conservation analysis.

### In Vitro Mutagenesis Experiments Confirm the Predictions of the Simulations

**Figure 6:**
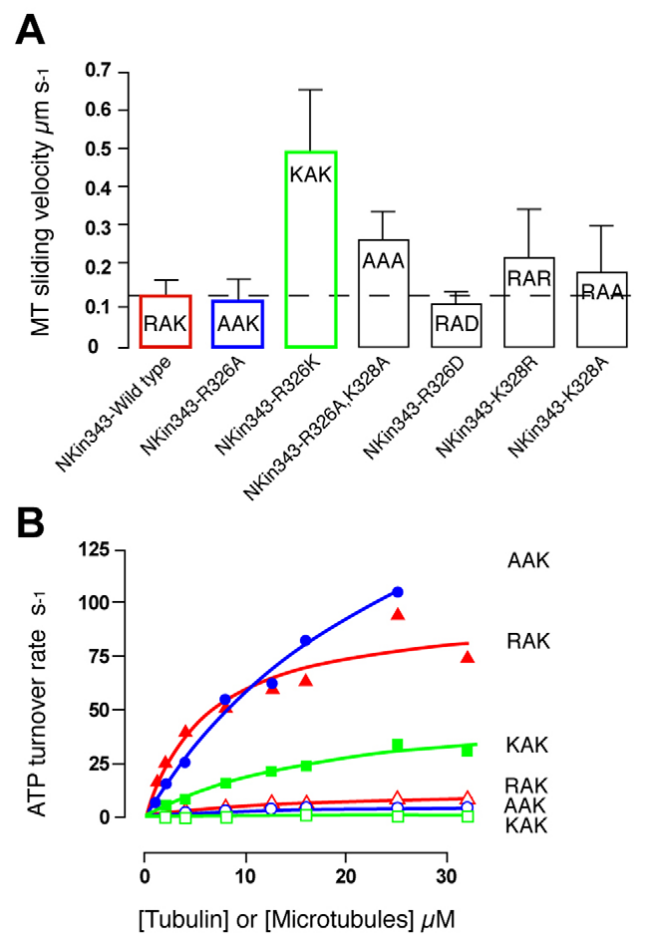
Experimental mutagenesis results. (A) Motility assay. Sliding velocity for R326A is not significantly different from wild type. By contrast, mutant R326K shows ∼5-fold increase in microtubule sliding velocity over wild type. (B) ATPase activation curves for tubulin and for microtubules of two key mutants AAK (R326A) and KAK (R326K) in Nkin343 monomeric kinesin-1. Mutant R326A shows a ∼2.5-fold increase in V_max_ for the microtubule-activated ATPase, with a ∼4-fold higher K_m_. Mutant R326K shows a modest decrease in V_max_ for microtubule-activation, with a 3-fold higher K_m_.

In tandem with our simulations, we performed in vitro experiments to test the effects of electrostatic mutations on kinesin function. Our computational analysis (Figure 5 and Table 1) identified charged residues predicted to have a profound effect on the on-rate of kinesin-1 to microtubules. Simulations also indicate that the distinct charge distribution of different kinesin sub-families can lead to a range of sub-family specific association rates (Figure 3A). To further probe the origin of these differences we focused on a three-residue segment at the C-terminus of helix α6. This region was observed to have a distinct sub-family-specific charge distribution in different kinesin sub-families (with a consensus sequence of RAK in subfamilies-1, -3, -5, and -13; SVN in kinesin-14; MTQ in kinesin-6 and RAR in kinesin-4). This region was previously shown to be essential for ATPase and motility [33] and was highlighted by both our electrostatic analysis and in another coarse-grained modeling study (Zheng et al., in prep). We made a series of experimental point mutants in NKin, a fast kinesin-1 from *Neurospora Crassa*, and assayed the effects of the mutations on microtubule sliding velocity, microtubule-activated ATPase, and tubulin-activated ATPase. A single-headed NKin construct was used, so as to mimic the conditions of the simulation. Tables 2 and S1 and Figure 6 summarize the results.

**Table 2:** The effects of selected RAK kinesin mutations on ΔG_elec_ and ΔΔG_elec_.

Mutation	ΔG_elec_* (kJ/mol)	ΔΔG_elec_ (kJ/mol)
KAK	−9.813	−2.2
RAR	−9.22	−1.607
RAK	−7.613	0
RAE	−6.819	0.794
RAD	−6.646	0.967
RAA	−6.506	1.107
AAA	−4.16	3.453
AAK	−3.677	3.936
DAK	−1.135	6.478

### Motility Assays and ATPase Assays Support a Key Role for the RAK Sequence

**Figure 7:**
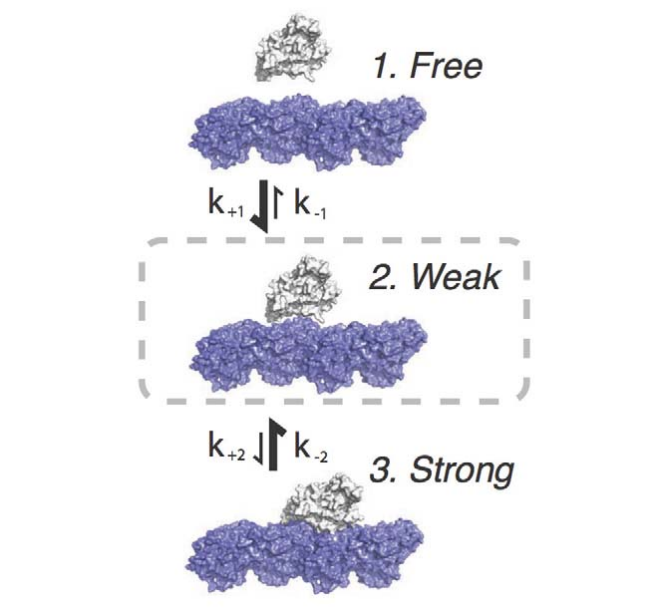
Kinetic scheme. In this 3-state scheme [34], mutagenesis that increases ΔΔG_elec_ will over-populate the weakly bound state (state 2) by enhancing recruitment from the free motor population (increasing k_+1_ and decreasing k_−1_) and from the strongly bound state (state 3) (by increasing k_−2_ and decreasing k_+2_). Increasing the population of state 2 relative to state 3 will decrease internal drag in the motility assay, thereby increasing microtubule sliding velocity.

All the mutants retained microtubule-activated and tubulin-activated ATPase activity. Both R321A (AAK) and the potentially more disruptive charge-reversal R321D (DAK) mutation are predicted by our simulations to have little effect, and the experiments confirm this. K323R (RAR) and K323A (RAA) are predicted to accelerate binding somewhat, and indeed increased microtubule sliding velocity 2-fold, compared to wild-type single head NKin. Replacing the RAK sequence with AAA resulted in a ∼3-fold velocity increase and R321K (KAK) produced a ∼5-fold increase in the velocity of kinesin-driven sliding microtubules. R321K does not affect the net charge on the molecule but does profoundly enhance the association of the motor to its microtubule track. Using purified pig brain tubulin (both as unpolymerized heterodimers and as microtubules, polymerized in the presence of Mg-GTP and taxol-stabilised), we measured the rates of microtubule-activated and tubulin-activated ATP hydrolysis and ADP release for wild-type and for RAK mutants (see Table S1). All constructs, wild type and mutant, were activated by free tubulin heterodimers, but to a lesser extent than by microtubules. For microtubule activation, the KAK mutant, which is 5-fold faster in motility assays, has a slightly reduced V_max_ in solution compared to wild type (∼54 s^−1^ compared to 97 s^−1^) and a ∼5-fold weaker apparent affinity for microtubules (Km ∼28 µM compared to 6 µM), The AAK mutant, which has wild type velocity in motility assays, also has a weaker apparent affinity for microtubules (K_m_ ∼19 µM) but shows an increased V_max_ (222 s^−1^). These results support a conventional model in which kinesin binds to microtubules in two steps, at first forming a “weak” state that attaches to microtubules but is not activated by them, and then shifting into a “strong” state that does show microtubule-stimulated product release [34]. Our simulations deal with the binding reaction that populates the initial, weakly bound state. We expect mutations that stabilize electrostatic interactions to accelerate the formation of this initial, weak binding state, and potentially also to accelerate exit from the strong state back into the weak state (Figure 7).

These dual effects over-populate the weak binding state, and this can account for the properties of our mutants in ATPase assays and motility assays. Microtubule sliding assays are accelerated because internal system drag, due to slowly detaching heads, is reduced. Microtubule-activated ATPase, averaged across the entire kinesin population, is little affected. We hypothesise that this is because the influence of faster initial formation of the weak-binding state is balanced by depopulation of the strong binding states (Figure 7).

Relating to Figure 7, we note that in order to explore electrostatic effects, we have treated the kinesin head as a rigid-body and focused exclusively on the diffusion-to-capture process. In future work we will aim to explore the role of electrostatics in the weak-to-strong conformational change and in subsequent steps in the mechanism.

### Conclusion

In summary, we find using atomistic Brownian dynamics simulations and in vitro mutational analysis that conserved electrostatic interactions enhance association and enable kinesin heads to preferentially bind tubulin heterodimers lying ahead in the progress direction. Furthermore, we find that the tethering of two heads in a dimer reduces the search space for binding sites on the microtubule lattice and further biases binding to a single microtubule protofilament. Simulations with different subfamily representatives and selected charge neutralizing mutations suggest that different kinesin subfamilies have tailored their electrostatic properties to modulate both their association rates and their directional bias along the microtubule. Taniguchi and colleagues [35] recently suggested that directional bias in walking kinesin dimers is predominantly entropic. It will be interesting to test this concept in relation to our proposal that directional electrostatically biased diffusional association is an intrinsic feature of the force-generating mechanism of kinesin minimal motor domains.

## Materials and Methods

Available kinesin crystal structures were obtained from the RCSB protein data bank and processed with the Bio3D package [36]. Processing involved initial extraction of motor domain coordinates corresponding to residues 9 to 325 in conventional kinesin-1. Subsequent alignment and superposition steps were as described in Grant et al. [26]. Missing regions of the various structures underwent standard molecular mechanics modeling and refinement protocols with the AMBER9 package [37]. Microtubule models were constructed by fitting multiple tubulin dimers to the 8 Å electron density map of Downing and coworkers [38].

### Electrostatic Calculations

Electrostatic calculations were performed with APBS (version 0.10.1) [39], using AMBER charges and radii at 310 K. Due to the high charge densities of the systems under consideration, the full, nonlinear Poisson–Boltzmann (PB) equation was solved in a multi-level fashion. Atomic charges were mapped to grid points via cubic B-spline discretization (chgm: spl2). The dielectric boundary between solute (with a dielectric constant of 4) and solvent (with a dielectric constant of 74) was specified as the van der Waals surface (srfm: mol and srad: 0).

### Electrostatic Similarity Analysis

Electrostatic potentials for available kinesin motor domain structures were analyzed with SurfaceDiver (version 1.0) [40]. Surface Diver employs spherical harmonic decomposition and a finite set of rotation-invariant descriptors to compare surface electrostatic properties. Based on these descriptors, molecules can be compared and clustered according to their electrostatic features without prior structural alignment. Operational parameters included a zero atom inflation radius (irad 0) and a maximal decomposition radius of 40 Å (rmax 40). Decomposition was performed on a total of 40 spherical surfaces (nsph 40) with a spherical harmonic decomposition order of 64 (spho 64). Complete-linkage hierarchical cluster analysis was performed with R and the Bio3D package.

### Brownian Dynamics

The BrownDye simulation package (version 1.0) [41] was employed for sub-family and mutant Brownian dynamics (BD) simulations. All atom models were used for both kinesin and tubulin. Because of uncertainties over the conformational dynamics of the neck linker, simulations used the head only (corresponding to residues 9–325 of kinesin-1, as for the electrostatics calculations above). Effective charges were used to reproduce pre-computed electrostatic potentials (see above). The influence of these potentials on the diffusional motion of both kinesin and tubulin was determined from the standard Ermak and McCammon algorithm [42]. Association rates were computed at 150 mM ionic strength with a modified version of the Luty, McCammon, and Zhou algorithm [43]. An adaptive time step with a minimum value of 1.0 ps was employed. Trajectories were propagated until the transient complex was obtained (see below) or until a center-to-center distance *c* (beyond *b*) was reached. Upon reaching *c* a pretabulated solution to the diffusion equation was used to determine whether the molecules would “escape” to infinity or return to some location with a center-to-center distance *b*. To obtain adequate statistics, 200,000–500,000 trajectories were simulated for each kinesin-tubulin pair.

The current version of BrownDye treats proteins as rigid bodies and does not take into account short-range interactions (van der Waals and hydrogen bonds). However, these interactions become important for short distances. Hence, the transition from encounter or transient complex to the subsequent bound states is beyond the realm of the current BD simulations and requires the application of more detailed models with explicit treatment of flexibility and short-range interactions.

### Defining the Transient Complex Boundary

As previously introduced, binding partners can be considered to pass through a transient intermediate state (A*B), in which the two proteins have near native separations and orientations. From this transient complex (also referred to as the encounter complex), non-diffusional rearrangements lead to the tightly bound native complex (AB).
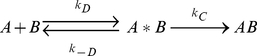
(1)Hence, the overall binding rate (*k_a_*) is given by:
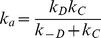
(2)The current BD simulations probe the diffusion-controlled rate (*k_D_*) for reaching the transient complex. In the transient complex, kinesin and tubulin must satisfy particular translational and rotational constraints. Defining these constraints provided a robust set of criteria for assessing successfully associated BD trajectories.

Initial atomic models for each kinesin-tubulin complex were built by fitting different kinesin crystal structures to a kinesin-tubulin complex obtained from a 9 Å CryoEM model of Moores and coworkers [25]. These complexes underwent molecular mechanics refinement with the AMBER9 package and corresponding all-atom potential function ff99SB (see Text S1). The resulting lowest energy models were used as the starting configurations for probing the bound state and the transition to the unbound state via the transient complex method of Zhou and coworkers [44].

The algorithm for identifying the transient complex boundary has been described in detail elsewhere. Briefly, to sample bound and unbound configurations, both kinesin and tubulin were treated as rigid. The kinesin motor domain was systematically translated and rotated with respect to the larger, fixed-in-space tubulin dimer. Steric clashes were monitored along with the number of inter subunit contacts (defined as heavy atoms having interfacial contacts less than 5 Å). For clash-free configurations, the number contacts (*Nc*) together with interface separation (*r*) and rotation angle (χ) were recorded (see Figure S3). The value of *Nc* (denoted as Nc*) at the onset of a sharp increase in χ was used to define the transient complex. These configurations (with *Nc* = Nc*) effectively separate the bound state, with numerous short-range interactions (high *Nc*) but restricted translational and rotational freedom (low *r* and χ), from the unbound state, with at most a small number of interactions (low *Nc*) but expanded configurational freedom (large *r* and χ).

### Mutational Analysis and Calculation of ΔG_elec_

Measuring the effects of mutations on the rate constants of association is a powerful tool to decipher the mechanism of association. Mutated residues were given a modeled conformation based on the most probable rotameric state and subsequent side-chain energy minimization with the AMBER9 package. BD simulations and the transient complex approach were used to examine the effect of a mutation on the association rates and binding affinities. As in previous studies, 100 configurations were randomly selected from the transient complex ensemble to calculate the average electrostatic interaction energy (ΔG_elec_) and the electrostatic interaction energy compared to wild-type (ΔΔG_elec_):
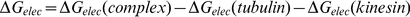
(3)

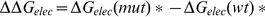
(4)where the two terms on the right side of equation 4 denote ΔG_elec_ after and before the mutation, respectively. For each transient complex configuration, ΔG_elec_ was calculated as described in equation 3. These results were then averaged to yield ΔG_elec_*. See Alsallaq et al. [44] for further details.

### Mutagenesis, Expression, and Purification of Proteins

Experiments used a 6xHistidine-tagged single-head NKin (6xHis-NKin343) as a starting construct, in which point mutations were created using PCR mutagenesis. Successful clones were verified by restriction site digestion and sequencing (Cogenics). The Histidine-tagged proteins were expressed in BL21/DE3 *E. coli* cells and purified using HisTrap Ni columns (GE Healthcare) using an AKTA Purifier system. Microtubule and tubulin-activated kinesin ATPase activities were measured using an enzyme-linked fluorescence assay [45], in a buffer (50 mM Pipes pH 6.9, 0.2 mM MgCl_2_, and 0.1 mM EGTA, 0.1 mg/ml BSA), at 25°C. For experiments involving microtubules Taxol was added to this buffer to a final concentration of 20 µM. K_d_ and V_max_ were determined by fitting the data to a hyperbola using Prism 4 for Macintosh. Motility assays were performed following the method described by Kaseda et al. [46]. Nitrocellulose-treated coverslips (0.1% nitrocellulose in isoamyl-acetate) were coated in penta-His antibody (Qiagen cat. No. 34660, diluted 1∶10 in PBS), incubated in a moisture chamber for 1 h, and then extensively washed with 1 mg/ml BSA in PBS to remove any unbound antibody. Histidine-tagged kinesin at 0.3–3 µM in assay buffer (50 mM Pipes pH 6.9, 0.2 mM MgCl_2_, 0.1 mM EGTA, 5 mM DTT, 20 µM Taxol, 0.2 mg/ml Casein, 1 mM ATP) was then flowed into the chamber and allowed to bind to the surface for 10 min. Unbound kinesin was washed away using assay buffer, taxol-stabilised microtubules introduced and allowed to bind for 10 min. Unbound microtubules were washed off with assay buffer containing the oxygen scavenger system [47] at 25°C. Control coverslips lacking antibody did not recruit microtubules from the overlying solution. Microtubule motility was recorded by video-enhanced DIC microscopy and quantified using the freeware RETRAC software (http://mechanochemistry.org/software). Motility assays were made in the same buffer conditions as the ATPase assays with the addition of 1 mM DTT and 0.1% casein.

## Supporting Information

Figure S1 Ionic strength dependence (I) of kinesin-1 association rates (k_D_) and electrostatic interaction energies (ΔG_elec_). See main text for details.
(TIF)

Figure S2 Additional results of kinesin-microtubule BD simulations. (A) Kinesin-14 monomer binding events. Each element of the table represents one of the 35 potential binding sites on the microtubule model and is labeled and colored by the proportion of binding events at the corresponding site (see main text and Figure 4 for further details). (B) Results of kinesin-1 monomer with a charge neutralized microtubule model.
(TIF)

Figure S3 Results of transient complex ensemble mapping of kinesin-1. The kinesin motor domain was systematically translated and rotated with respect to the larger, fixed-in-space tubulin dimer. Steric clashes were monitored along with the number of inter subunit contacts (defined as heavy atoms having interfacial contacts less than 5 Å). For clash-free configurations the number of contacts (Nc) together with interface separation (r) and rotation angle (χ) are plotted in (A) and (B), respectively. (C) The value of Nc at the onset of a sharp increase in σχ (denoted as Nc* in the main text and marked with a dashed blue line in (A–C)) was used to define the transient complex boundary. (D) Representative configurations in the transient complex (6).
(TIF)

Figure S4 Kinesin-tubulin association. Center-of-mass distance (black line) versus relative torsion angle (gray line) between kinesin and tubulin during a successful approach trajectory at 250 mM ionic strength. Compare to Figure 3C and see main text for details.
(TIF)

Movie S1 Surface mapped electrostatic potentials of the kinesin family. Values are expressed as a color spectrum ranging from +5 kT/e (blue) through 0 kT/e (white) to −5 kT/e (red). Panels correspond to front (toward the nucleotide binding site), rear, and mid-sliced views of the motor domain. Note, despite the overall diversity in charge distribution, the consistent positive patch (blue) on the rear face of the motor domain (see also Movie S2).
(MOV)

Movie S2 Consensus electrostatic potential map of the kinesin family. Illustrating the percentage of structures having a potential of the same sign at a particular region of space. Consensus potentials are displayed at the 80% level with a transparent surface and the 100% level with a solid surface, see also Movie S1.
(MOV)

Movie S3 A typical Brownian dynamics simulation. The simulation is initiated with kinesin and tubulin in random orientations and positions on the “initiation sphere,” where electrostatic energy contours are centrosymmetric. At large distances both proteins will undergo free diffusion leading to possible “escape.” At closer distances each protein will start to experience the electrostatic field of the other protein. Eventually, kinesin and tubulin will be close enough to favorably orient themselves with respect to their electrostatic fields. Note that in the simulations, both proteins are freely diffusing; here, for clarity, the camera tracks around the tubulin heterodimer.
(MOV)

Table S1 Effects of select RAK kinesin mutations on K_d_ and V_max_.
(DOC)

Text S1 Molecular mechanics refinement of transient complex models.
(DOC)
