# Supplementary figures and images for: Electrostatically Biased Binding of Kinesin to Microtubules
Source: PLoS Biol. 2011 Nov 29;9(11):e1001207. doi: 10.1371/journal.pbio.1001207 (PMC3226556; doi:10.1371/journal.pbio.1001207)

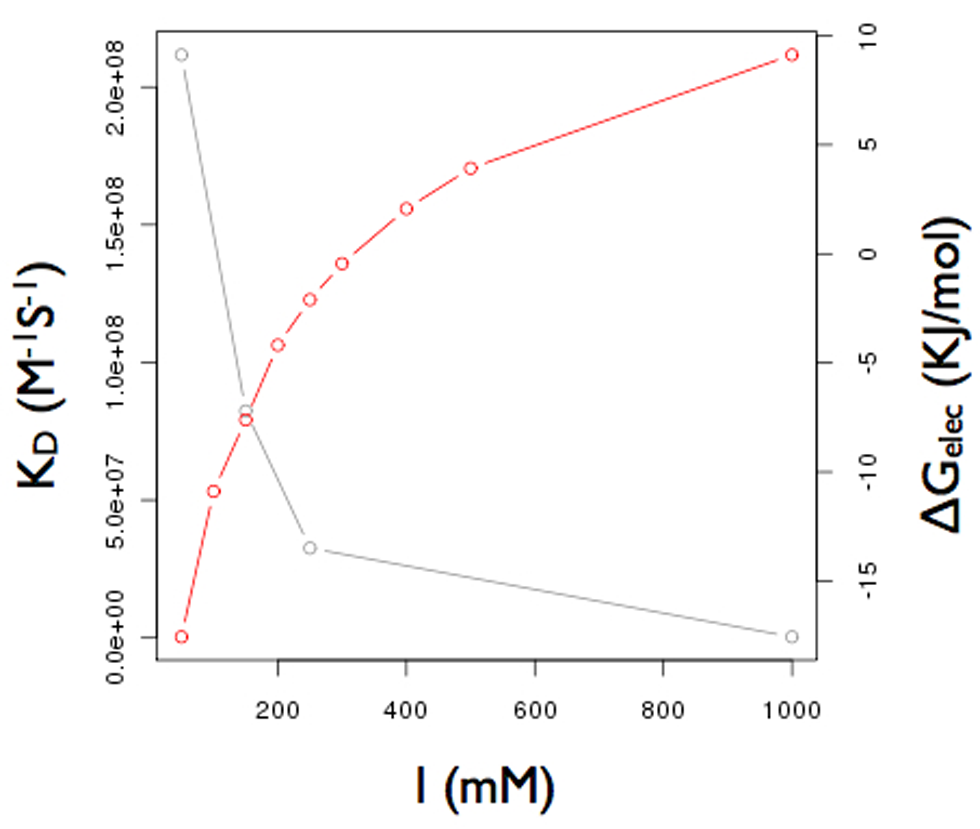

Supplement: Figure S1 — Ionic strength dependence (I) of kinesin-1 association rates (kD) and electrostatic interaction energies (ΔGelec). See main text for details. (TIF) [file pbio.1001207.s001.tif]

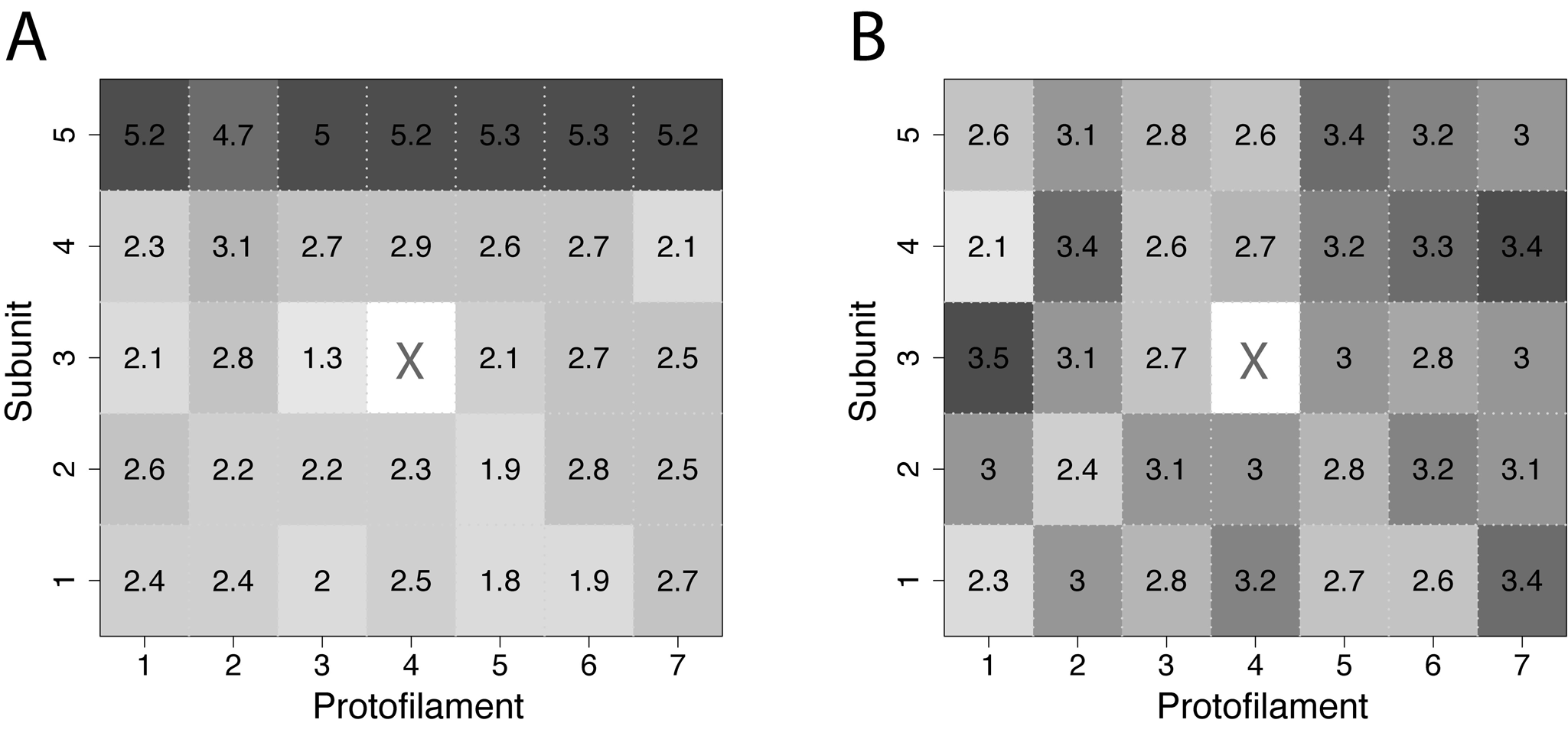

Supplement: Figure S2 — Additional results of kinesin-microtubule BD simulations. (A) Kinesin-14 monomer binding events. Each element of the table represents one of the 35 potential binding sites on the microtubule model and is labeled and colored by the proportion of binding events at the corresponding site (see main text and Figure 4 for further details). (B) Results of kinesin-1 monomer with a charge neutralized microtubule model. (TIF) [file pbio.1001207.s002.tif]

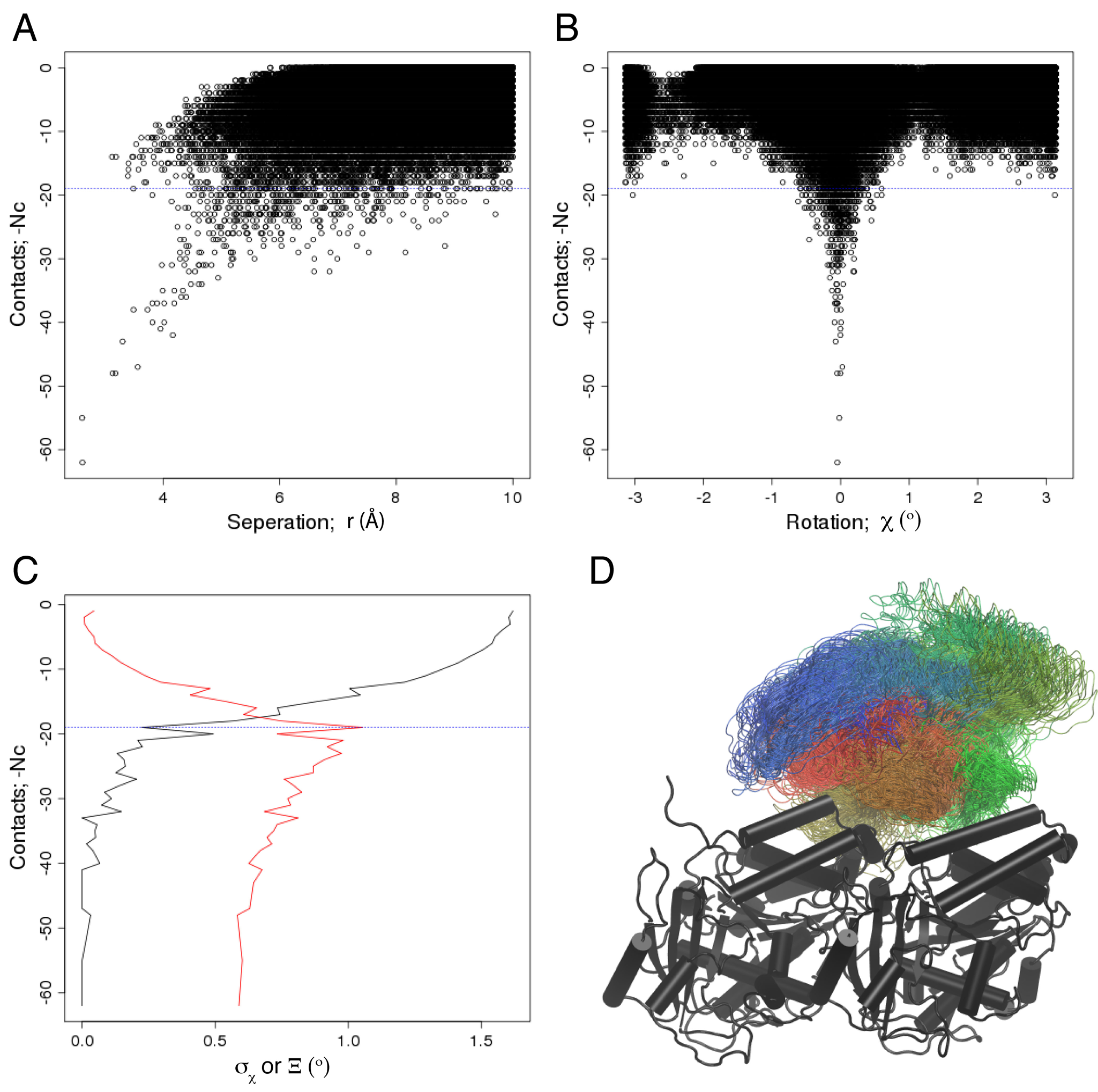

Supplement: Figure S3 — Results of transient complex ensemble mapping of kinesin-1. The kinesin motor domain was systematically translated and rotated with respect to the larger, fixed-in-space tubulin dimer. Steric clashes were monitored along with the number of inter subunit contacts (defined as heavy atoms having interfacial contacts less than 5 Å). For clash-free configurations the number of contacts (Nc) together with interface separation (r) and rotation angle (χ) are plotted in (A) and (B), respectively. (C) The value of Nc at the onset of a sharp increase in σχ (denoted as Nc* in the main text and marked with a dashed blue line in (A–C)) was used to define the transient complex boundary. (D) Representative configurations in the transient complex (6). (TIF) [file pbio.1001207.s003.tif]

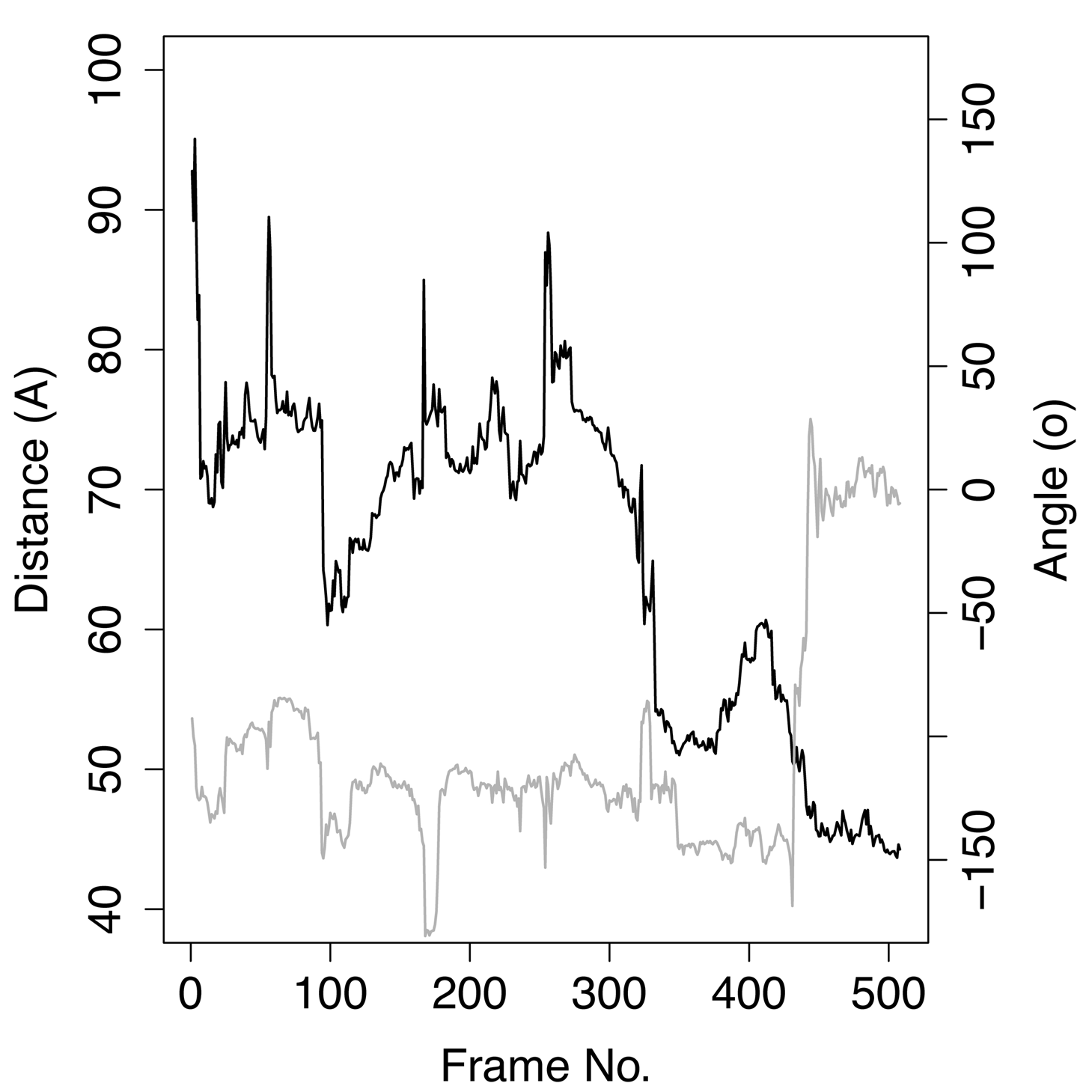

Supplement: Figure S4 — Kinesin-tubulin association. Center-of-mass distance (black line) versus relative torsion angle (gray line) between kinesin and tubulin during a successful approach trajectory at 250 mM ionic strength. Compare to Figure 3C and see main text for details. (TIF) [file pbio.1001207.s004.tif]
