# Supplementary material for: Electrostatically Biased Binding of Kinesin to Microtubules
Source: PLoS Biol. 2011 Nov 29;9(11):e1001207. doi: 10.1371/journal.pbio.1001207 (PMC3226556; doi:10.1371/journal.pbio.1001207)
Supplement: Table S1 — Effects of select RAK kinesin mutations on Kd and Vmax. (DOC) [file pbio.1001207.s008.doc]

**Electrostatically biased binding of kinesin to microtubules.**

Barry J. Grant^a^,*, Dana Gheorghe^c^,*, Wenjun Zheng^d^, Maria Alonso^c^, Gary Huber^a^, Maciej Dlugosz^e^, J.Andrew McCammon^ab†^ and Robert A. Cross^c†^

^a^ Department of Chemistry and Biochemistry, Center for Theoretical Biological Physics and Howard Hughes Medical Institute, University of California San Diego, La Jolla, California, 92093, USA.

^b^ Department of Pharmacology, University of California San Diego, La Jolla, California, 92093, USA.

^c^ Centre for Mechanochemical Cell Biology, Warwick Medical School, University of Warwick, Coventry, CV4 7AL, UK.

^d^ Department of Physics, University at Buffalo, Buffalo, New York, 14260-1500, USA

^e^ Interdisciplinary Centre for Mathematical and Computational Modelling, University of Warsaw, Zwirki i Wigury 93, 02-089, Warsaw, Poland.

^f^ Present address: Center for Computational Medicine and Bioinformatics, University of Michigan, 100 Washtenaw Avenue, Ann Arbor, Michigan 48109-2800, USA

* Denotes equal contribution.

† Denotes joint senior authors.

Correspondence should be addressed to R.A.C *email: rob@mechanochemistry.org*

and B.J.G. *email*[*: bjgrant@umich.edu*](mailto:: bjgrant@umich.edu)

**Supporting Material**

*Tables*

**Table S1.** The effects of select RAK kinesin mutations on K_d_ and V_max_, see Fig. 5 and main text for details.

|  | Brain tubulin | | Brain microtubules | | Calculated **ΔΔG_elec_ (kJ/mol)** | |
| --- | --- | --- | --- | --- | --- | --- |
|  | **K_d_** | **V_max_** | **K_d_** | **V_max_** | **ΔG_elec_** | **ΔΔG_elec_** |
| NKin343-6xHis  (single head WT) | 16.5±1.3 | 13.3±0.5 | 6.1±2.0 | 96.8±10.1 |  | 0 |
| Nkin343-R326A-6xHis | 8.3±2.0 | 5.2±0.4 | 27.9±5.9 | 222.5±29.8 | ???? | ???? |
| Nkin343-R326K-6xHis | 8.0±2.1 | 1.2±0.1 | 18.7±4.6 | 53.4±6.5 | -9.813 | -2.2 |
