# Supplementary material for: Electrostatically Biased Binding of Kinesin to Microtubules
Source: PLoS Biol. 2011 Nov 29;9(11):e1001207. doi: 10.1371/journal.pbio.1001207 (PMC3226556; doi:10.1371/journal.pbio.1001207)
Supplement: Text S1 — Molecular mechanics refinement of transient complex models. (DOC) [file pbio.1001207.s009.doc]

**Electrostatically biased binding of kinesin to microtubules.**

Barry J. Grant^a^,*, Dana Gheorghe^c^,*, Wenjun Zheng^d^, Maria Alonso^c^, Gary Huber^a^, Maciej Dlugosz^e^, J.Andrew McCammon^ab†^ and Robert A. Cross^c†^

^a^ Department of Chemistry and Biochemistry, Center for Theoretical Biological Physics and Howard Hughes Medical Institute, University of California San Diego, La Jolla, California, 92093, USA.

^b^ Department of Pharmacology, University of California San Diego, La Jolla, California, 92093, USA.

^c^ Centre for Mechanochemical Cell Biology, Warwick Medical School, University of Warwick, Coventry, CV4 7AL, UK.

^d^ Department of Physics, University at Buffalo, Buffalo, New York, 14260-1500, USA

^e^ Interdisciplinary Centre for Mathematical and Computational Modelling, University of Warsaw, Zwirki i Wigury 93, 02-089, Warsaw, Poland.

^f^ Present address: Center for Computational Medicine and Bioinformatics, University of Michigan, 100 Washtenaw Avenue, Ann Arbor, Michigan 48109-2800, USA

* Denotes equal contribution.

† Denotes joint senior authors.

Correspondence should be addressed to R.A.C *email: rob@mechanochemistry.org*

and B.J.G. *email*[*: bjgrant@umich.edu*](mailto:: bjgrant@umich.edu)

**Supporting Material**

*Methods:*

*Molecular mechanics refinement of transient complex models*

Initial atomic models for each kinesin-tubulin complex were built from fitting different kinesin crystal structures to a kinesin-tubulin complex obtained from a 9Å CryoEM model of Moores and coworkers (1). These complexes underwent molecular mechanics refinement (minimization and molecular dynamics) with the AMBER9 package and corresponding all-atom potential function ff99SB (2, 3). Additional parameters for nucleotides were taken from Meagher et al. (4). The LEaP module was used for model construction with each kinesin model simulated in the presence of Mg2+·ADP. A modified generalized Born model (GB^OBC^) was used to describe solvation effects (5). Energy minimization with decreasing constraints on heavy atom positions was followed by unconstrained minimization. Constant volume heating (to 300 K) was carried out over 10 ps, followed by constant temperature (300 K) and constant pressure (1 atm) equilibration for additional 200 ps. Operational parameters included a 2fs time step and a 10Å cutoff for the truncation of VDW non-bonded interactions. The resulting models were used as the starting configurations for probing the bound state and the transition to the unbound state via the transient complex method of Zhou and coworkers (6).

*References*

1. Bodey, A. J., M. Kikkawa, and C. A. Moores. 2009. 9-Angstrom structure of a microtubule-bound mitotic motor. J Mol Biol 388:218-224.

2. Case, D. A., T. E. Cheatham, 3rd, T. Darden, H. Gohlke, R. Luo, K. M. Merz, Jr., A. Onufriev, C. Simmerling, B. Wang, and R. J. Woods. 2005. The Amber biomolecular simulation programs. J Comput Chem 26:1668-1688.

3. Hornak, V., R. Abel, A. Okur, B. Strockbine, A. Roitberg, and C. Simmerling. 2006. Comparison of multiple Amber force fields and development of improved protein backbone parameters. Proteins 65:712-725.

4. Meagher, K. L., L. T. Redman, and H. A. Carlson. 2003. Development of polyphosphate parameters for use with the AMBER force field. J Comput Chem 24:1016-1025.

5. Onufriev, A., D. Bashford, and D. A. Case. 2004. Exploring protein native states and large-scale conformational changes with a modified generalized born model. Proteins 55:383-394.

6. Alsallaq, R., and H. X. Zhou. 2008. Electrostatic rate enhancement and transient complex of protein-protein association. Proteins 71:320-335.
